# Supplementary figures and images for: An in vivo drug screen in zebrafish reveals that cyclooxygenase 2‐derived prostaglandin D2 promotes spinal cord neurogenesis
Source: Cell Prolif. 2023 Dec 28;57(5):e13594. doi: 10.1111/cpr.13594 (PMC11056714; doi:10.1111/cpr.13594)

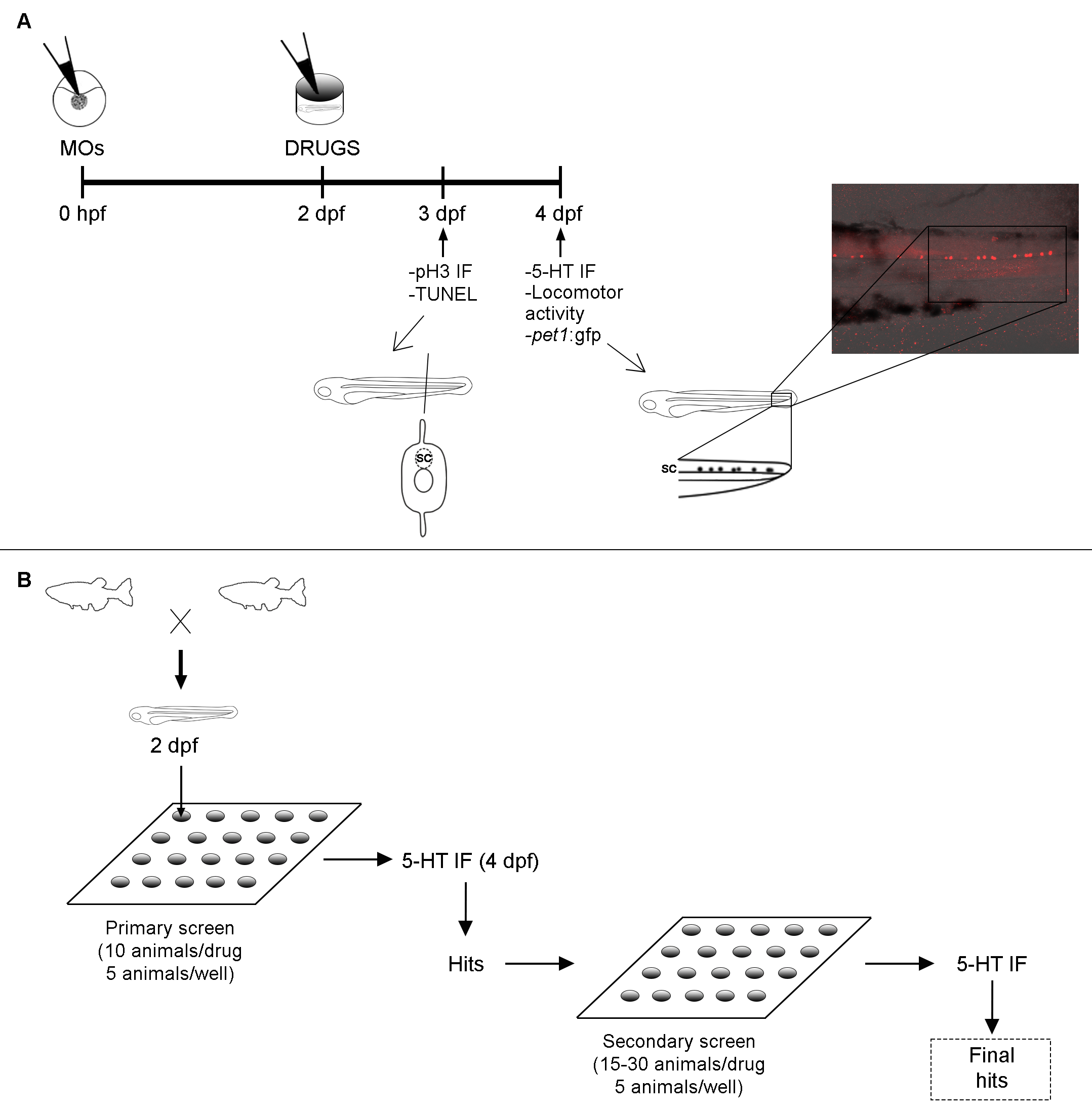

Supplement: Supplementary file 1 — Supplementary Figure 1. (A) Schematic drawing showing the experimental design and morpholino and drug treatments' time windows. (B) Schematic of the drug screening protocol. Abbreviations: IF, immunofluorescence; MOs, morpholinos; SC, spinal cord. [file CPR-57-e13594-s001.tif]

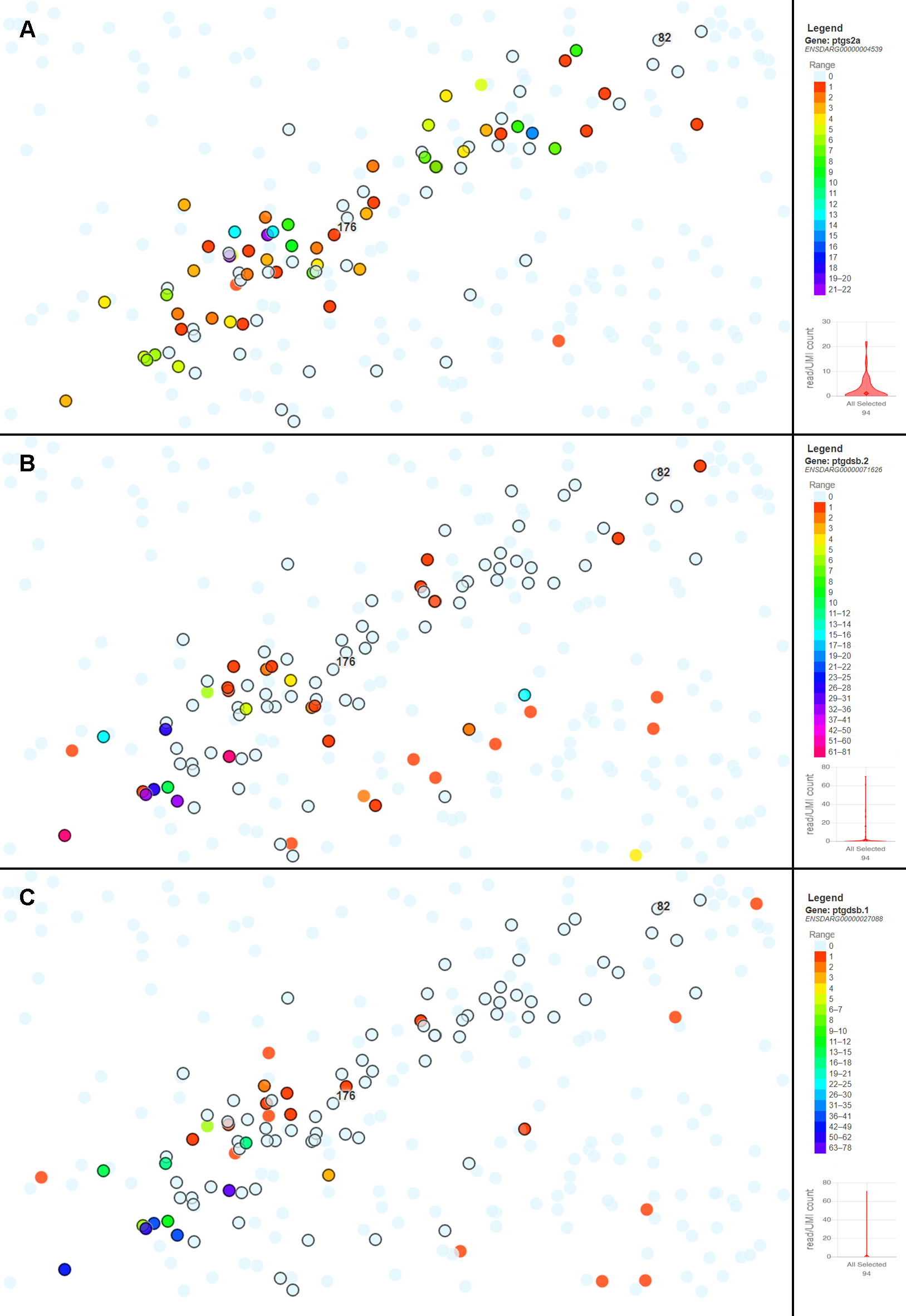

Supplement: Supplementary file 2 — Supplementary Figure 2. Graphs from the scRNAseq atlas (each individual cell is indicated by a light blue dot) of developing zebrafish 7 showing the expression of ptgs2a (A), ptgdsb.2 (B) and ptgdsb.1 (C) in FP cells (cluster 176; cells of this cluster are indicated by black circles). FP cells expressing these genes are colour coded according to the legends on the right. [file CPR-57-e13594-s004.tif]
